# Deep Eutectic Solvents-Based Ultrasound-Assisted Extraction of Antioxidants from Kudingcha (*llex kudingcha* C.J. Tseng): Process Optimization and Comparison with Other Methods

**DOI:** 10.3390/foods12091872

**Published:** 2023-04-30

**Authors:** Fangliang Li, Leyan Xiao, Xue Lin, Jincheng Dai, Jiale Hou, Lu Wang

**Affiliations:** 1 School of Food Science and Engineering, Hainan University, Haikou 570228, China; 2 Key Laboratory of Food Nutrition and Functional Food of Hainan Province, Hainan University, Haikou 570228, China

**Keywords:** *llex kudingcha* C.J. Tseng, deep eutectic solvents, bio-active compounds, antioxidant activity, response surface methodology

## Abstract

Kudingcha (KDC) is an important tea substitute containing abundant antioxidants. Herein, a ultrasonic-assisted extraction (UAE) technique based on deep eutectic solvents (DESs) was applied to optimize the total phenolic/total flavonoid content (TPC/TFC) from the KDC extracts. Results indicated that DES composed of L-proline and glycerol (Pro-Gly) had excellent extraction performance for TPC, TFC, ABTS^•+^ and FRAP, which were significantly better than other solvents. Response surface methodology (RSM) was used to obtain optimal extraction parameters for simultaneously maximizing the TPC, TFC and antioxidant activity. Results revealed that water content in Pro-Gly, liquid to solid ratio (L/S), ultrasonic temperature and extraction time were the major influence factors of the TPC, TFC, ABTS^•+^ and FRAP of the KDC extracts. The optimal conditions included water content in Pro-Gly of 46.4%, L/S of 25:1 (mL/g), ultrasonic temperature of 55 °C and extraction time of 50 min. Meanwhile, HPLC-MS/MS was adopted to identify the KDC extracts, which revealed the presence of major phytochemicals, including 5-chlorogenic acid, 4,5-dicaffeoylquinic acid, 3,5-dicaffeoylquinic acid, 3,4-dicaffeoylquinic acid, kaempferol 3-rutinoside, myricetin and isorhamnetin. Moreover, UAE–Pro-Gly achieved further higher individual phenolics contents, TPC, TFC, ABTS^•+^ and FRAP than other methods. In conclusion, UAE–Pro-Gly is a highly efficient method for extraction of phenolic antioxidants from KDC.

## 1. Introduction

Kudingcha Holly is an evergreen tree plant of genus *Ilex chinensis* Sims, commonly known as Kudingcha, Fudingcha and Gaolu tea, mainly distributed in southwest China [1]. It can be roughly divided into three species, namely, *Ilex kudingeha* C.J. Tseng, *Ilex latifolia* Thunb and *Ilex cornuta* Lindl. Ex Paxt. [2]. In China, Kudingcha (*llex kudingcha* C.J. Tseng) has been considered as a substitute tea or Chinese herbal medicine for more than two thousand years [3,4,5]. A lot of scientific research has confirmed that Kudingcha (KDC) extract possessed various health-related benefits and neuroprotection effects [6,7,8]. In addition, the secondary metabolites of the KDC extract, including phenolics, coumarins, flavonoids, and polysaccharides, etc., are positively associated with human health [9]. Meanwhile, phenolics are key secondary metabolites in Kudingcha and have extensive biological activities, which causes Kuding tea to have good medicinal values and clinical application [8,9]. Many researchers have tried to exploit natural antioxidants instead of synthetic compounds [10,11]. On this basis, it is urgent to develop an efficient extraction method to obtain natural antioxidants from Kudingcha.

At present, water, methanol, acetone, ethanol and ethyl acetate are major solvents traditionally used to extract antioxidants from natural products [12]. However, these solvents have some disadvantages. In addition, most organic solvents are volatile, flammable, toxic and not easy to degrade, so they are not suitable to be used in the food and pharmaceutical industries [13]. Recently, natural deep eutectic solvents (DESs) have attracted more and more attention [14]. DES is a low melting point mixture formed by a hydrogen bond acceptor (HBA) and a hydrogen bond donor (HBD) under heating conditions. This concept was first proposed by Abbott et al. in 2003 [15]. Not only do DES have excellent characteristics such as low toxicity or no toxicity, low volatility, wide polarity, easy bio-degradation, high solute stability and solubility, but it has also become the best substitute for traditional organic solvents with benefits including: low price, easy synthesis and efficient recycling [16]. On the other hand, extraction methods have a major impact on active ingredients extraction [17]. Currently, compared with conventional extraction techniques, DES-based ultrasound-assisted extraction (UAE) reduces solvent and energy consumption, but damages plant cell walls through the cavitation effect caused by ultrasonic processing. By releasing more bio-active substances and reducing the diffusion boundary layer, the energy transfer of the solvent system can be enhanced, and the extraction efficiency of active ingredients is improved [17,18]. As of now, the extraction of natural antioxidants from Kudingcha using DES-based UAE has rarely been reported.

This study aims to extract the natural antioxidants from KDC by using DES combined with an ultrasound method. A high-efficiency solvent was used for the extraction of antioxidants from KDC for the first time, and the characterization of DES was analyzed. Subsequently, the process parameters of DES-based UAE were optimized using RSM. The phenolics of the KDC extracts were identified using HPLC-MS/MS. The study provides a novel method to enhance the extraction of natural antioxidants from Kuding tea. 

## 2. Materials and Methods

### 2.1. Materials

Kudingcha (KDC) leaves were purchased from Hainan Yexian Biotechnology Co.; Ltd.(Hainan, China) The leaves were first freeze-dried for 40 h in a LGJ-10 type vacuum freeze-dryer (Songyuanhuaxing Technology Develop Co., Ltd., Beijing, China) and then ground into powders by DFT-50A mill and subjected to a 40-mesh sieve. Folin-Ciocalteu reagent, 6-hydroxy-2,5,7,8-tetramethylchroman-2-carboxylic acid (Trolox, >99.8%), 2-Azino-bis (3-ethylbenzothiazoline-6-sulfonic acid) diammonium salt (ABTS, >99.7%), 2,2-diphenyl-1-picrylhyldrazyl radical (DPPH, >99.7%) and 2,4,6-Tris(2-pyridyl)-s-triazine (TPTZ, >99.8%) were provided by Sigma-Aldrich, China. All phenolic standards (HPLC-grade, >99.8%) were purchased from Nanjing Herbal Origin Biotechnology Co., Ltd.(Nanjing, China) Acetonitrile and formic acid in HPLC-grade were bought from Fisher Scientific. Chemicals for DES preparation were purchased from Aladdin Chemical Co. Ltd., (Shanghai, China). Other reagents were purchased from Xilong chemical Co., Ltd. (Guangzhou, China).

### 2.2. Preparation and Physicochemical Properties of DESs

**Figure 1:**
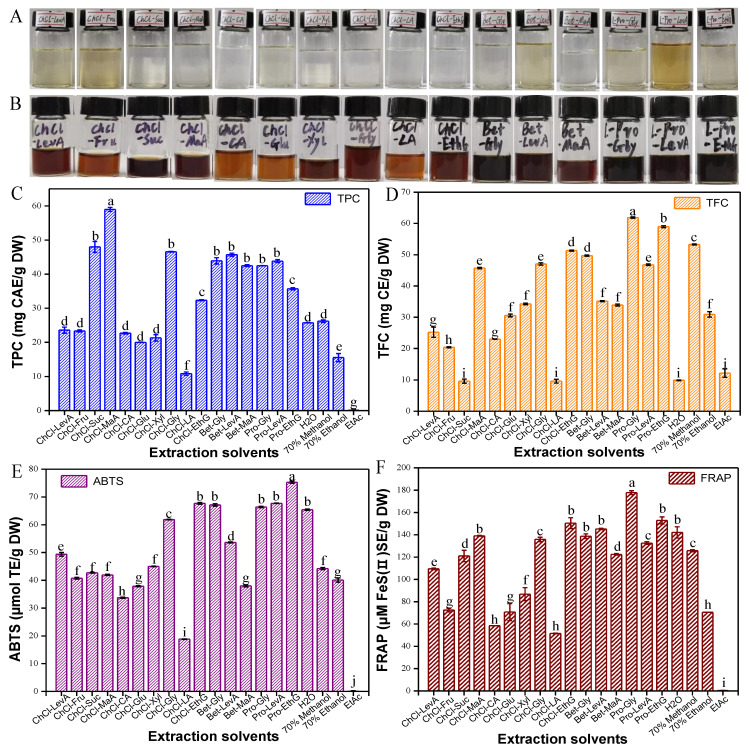
The visual appearance of the prepared DESs (**A**), and the visual appearance (**B**), TPC (**C**), TFC (**D**), ABTS^•+^ (**E**) and FRAP (**F**) of the KDC extracts obtained by DESs. Different lowercase letters (a–i) mean statistically significant differences in TPC, TFC, ABTS^•+^ and FRAP of the KDC extracts.

In this study, sixteen types of DESs were prepared using the heating and stirring method proposed by Wang et al. [19]. HBAs and HBDs were mixed at appropriate molar ratios and added to a sealed flask equipped with a magnetic stir bar; the mixture was heated and stirred at 80 °C until a stable clear liquid was formed (Table 1 and Figure 1A) [19]. The viscosity of the DESs prepared was determined using a HAAKE MARS 40 type rheometer (ThermoFisher Scientific, Karlsruhe, Germany) [16]. According to the results of our previous experiments, adding 30% ultrapure water (*w*/*w*) into the prepared DESs led to the reduction of DES viscosity, further enhancing the extraction efficiency for active compounds. The polarity of DESs prepared was measured using Nile red as the solvatochromic probe according to the method of Huang et al. [16]. 

### 2.3. Extraction of Bio-Active Compounds from KDC

The dried KDC powder (0.5 g) was placed into 5 mL of the prepared DES in a 10 mL tube. Then, the mixture was extracted under ultrasonic power of 320 W for 30 min at 40 °C, followed by centrifugation treatment at 10,000× *g* for 10 min to collect the supernatants. They were stored at 4 ℃ for the next experiments.

### 2.4. Determination of TPC and TFC

Total phenolic content (TPC) was examined by Folin–Ciocaltue reagent approaches, with slight modification [20]. Briefly, 100 μL of the diluted KDC extract was mixed with 300 μL of Folin–Ciocalteu reagent in a 2 mL tube for incubation at 30 °C for 5 min. Then, 450 μL of 20% Na_2_CO_3_ solution was added into the tube for incubation at 30 °C for 15 min under dark conditions. Finally, 200 μL of the above mixture was added to a 96-well microplate. Next, the absorbance at 747 nm was recorded by a microtiter plate reader (Molecular Devices, MA, USA). A regression curve was drawn using 5-chlorogenic acid as standard, and TPC was expressed as mg CAE/g DW, where CAE represents 5-chlorogenic acid equivalents.

Total flavonoid content (TFC) was tested using the AlCl_3_ protocol, with slight modification [17]. First, 100 μL of the KDC extract, 100 μL of methanol and 50 μL of 5% NaNO_2_ solution was mixed in a 2 mL Eppendorf tube, and they were allowed to react at 30 °C for 5 min. After that, 50 μL of the 10% AlCl_3_ solution was added for incubation for another 6 min. Finally, 400 μL of 1 M NaOH solution was added to terminate the reaction. After incubation for 15 min at 30 °C, the absorbance at 510 nm was recorded. The calibration curve was plotted using rutin as the standard, and TFC was expressed as mg RE/g DW, where RE represents rutin equivalents. 

### 2.5. Antioxidant Activity

ABTS^•+^ radical scavenging activity was investigated according to the previously described method [21]. The regression curve was plotted using Trolox as the standard, and the results were expressed as mmol TE/g DW, where TE represents Trolox equivalents.

Ferric reducing antioxidant power (FRAP) was investigated as well [22], and the FRAP results were expressed as mM Fe(II)SE/g DW, where SE represents sulphate equivalents.

### 2.6. Experimental Design

#### 2.6.1. Full-Factorial Design (FFD) Experiment

A FFD experiment was carried out [19]. The distribution matrix was constructed using Design Expert 10.0 software. The TPC, TFC, ABTS^•+^ and FRAP of the KDC extracts were comprehensively investigated by taking water content in DES, ultrasonic power, ultrasonic temperature, ultrasonic time and L/S ratio as variables (Table S1). The key influencing factors were selected for further optimization. Each of the experiments was conducted in triplicate.

#### 2.6.2. Response Surface Methodology (RSM) 

On the basis of the FFD experiment, the extraction temperature (A, 10–40–70 °C), extraction time (B, 5–35–65 min), L/S (C, 10–20–30 mL/g) and water content in Pro-Gly (D, 10–40–70%) were considered as the major influencing variables [18]. Table 1 shows the coded levels of the independent variables, and their influences on Y_TPC_, Y_TFC_, Y_ABTS_, and Y_FRAP_ were investigated by a 30 CCD experiment. The coefficients of regression equation were evaluated using Design Expert 10.0 Trial software. An empirical model was obtained by multiple regression analysis. Table 2 shows the comparison of measured and predicted results. In the RSM experiment, the second-order response function was predicted by Equation (1).

(1) $$Y=\beta_{0}+\sum_{i=1}^{n}\beta_{i}X_{i}+\sum_{\begin{array}{l} i=1\\ j>1 \end{array}}^{n=1}\sum_{j=2}^{n}\beta_{ij}X_{i}X_{j}+\sum_{i=1}^{n}\beta_{ii}X_{i}^{2}+\varepsilon$$

where Y_TPC_, Y_TFC_, Y_ABTS_, and Y_FRAP_ are the responses, *X_i_* and *X_j_* are independent variables, *β_0_, β_i_, β_ii_* and *β_ij_* are the intercept term, coefficients, quadratic coefficients and coefficients of interaction effects, respectively. ε is a random error.

### 2.7. Comparison of Different Extraction Methods

#### 2.7.1. Heating Extraction (HE)

First, 0.3 g of the dried KDC powder was mixed with 7.5 mL of water, MeOH, or Pro-Gly (46% water content) at a ratio of 25:1 (mL/g), and then, they were heated in a XMTD-204 thermostat water bath at 55 °C for 50 min, followed by centrifugation (10,000× *g*, 10 min) to collect supernatants.

#### 2.7.2. Microwave-Assisted Extraction (MAE) 

First, 0.3 g of the KDC powder and 7.5 mL of Pro-Gly (46% water content) were mixed at a ratio of 25:1 (mL/g). Then, extraction was conducted in a NN-GF37JW microwave oven at 400 W for 30 s before carrying out centrifugation at 10,000× *g* for 10 min to collect supernatants. 

#### 2.7.3. Ultrasound-Assisted Extraction (UAE)

In this experiment, 0.3 g of the KDC powder was mixed with 7.5 mL of Pro-Gly (46% water content) in 10 mL centrifuge tubes. Then, extraction was conducted under the optimal RSM conditions before centrifugation at 10,000× *g* for 10 min to collect supernatants. 

### 2.8. Chemical Compositions Analysis

All the KDC extracts were filtered using a 0.22 μm syringe filter for injection in an Agilent 1260 HPLC system coupled with a diode array detector (DAD) and an electrospray ionization mass spectrometer (ESI-MS) (Bruker, MA, USA). Separation was performed in a Zorbax SB C18 plus column. Elution was made with phase A (0.1% HCOOH-CH_3_CN) and phase B (0.1% HCOOH-water). The following elution gradient was used: 0−5 min, 15% B; 5−25 min, 25−35% B; 25−40 min, 25−50% B; 40−45 min, 85% B; and 45–50 min, 15% B. The ESI-MS conditions were consistent with those in the study of Wang et al. [23]. Bruker Daltonics Data Analysis software was adopted to process the data. The major phenolics were quantified by the HPLC-DAD method, and the chromatographic conditions were in agreement with the HPLC-ESI-MS/MS method. 

### 2.9. SEM

The morphology of the samples was revealed using a SEM (JSM-7610FPlus, Tokyo, Japan). After extraction, all the sample residues were washed three times with distilled water and then vacuum freeze-dried. Finally, the untreated KDC raw and sample residues extracted were gold plated and photographed by scanning electron microscope.

### 2.10. Statistical Analysis

Data were processed using IBM SPSS Statistics Version 19.0, and the results were expressed in the form of mean value ± SD. RSM experiments were performed using Design Expert software version 10.0 (Stat-Ease Inc., Minneapolis, MN, USA). The quality of the fitted model CCD was evaluated by ANOVA, and differences were significant when *p* < 0.05. 

## 3. Results and Discussion

### 3.1. Physicochemical Properties of DESs

**Figure 2:**
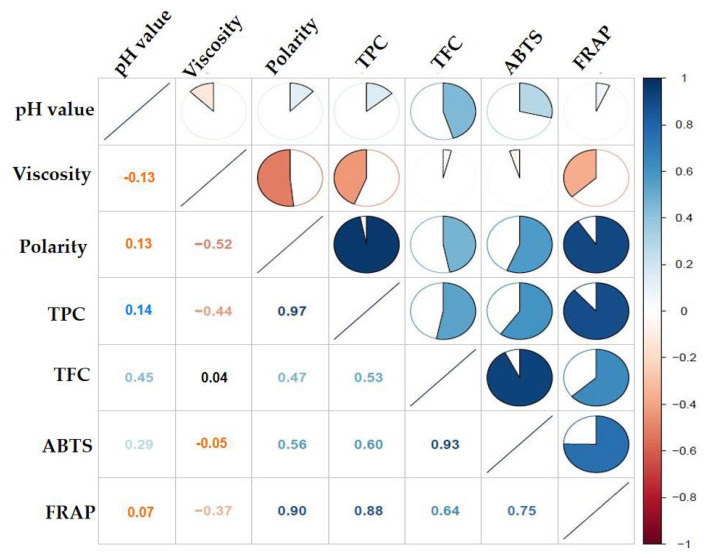
The correlation coefficient of the physical-chemical properties of DESs and the corresponding TPC, TFC, ABTS^•+^ and FRAP.

The extraction efficiency of phenolic/flavonoid compounds using acid-based DESs, choline chloride-based DESs and alcohols-based DESs is better than that using sugar-based DESs [24]. In this study, betaine, choline chloride and L-proline as HBAs as well as three types of HBDs were adopted to prepare DESs. As depicted in Figure 1A, all DESs prepared were stable, homogeneous and transparent. After KDC was extracted with the 16 types of DESs, the extracts displayed a darker color, which was significantly different from the primary colorless DESs. (Figure 1B). Researchers have reported that DESs played an important role in the solubilization, transport and release of poorly water-soluble metabolites in plant cellular tissues [24]. In the present work, the effects of DESs and conventional solvents on TFC, TPC, ABTS^•+^ and FRAP of the KDC extracts were investigated (Figure 1C,D). It was found that the TPC and TFC of the KDC extracts were significantly affected by solvents. ChCl-MA brought the highest extraction yield of TPC and Pro-Gly, which led to the highest extraction yield of TFC. ChCl-Gly and Pro-EthG exhibited solid extraction efficiency for TPC and TFC. The TPC extracted by water was consistent with that extracted by 70% methanol, but TFC extracted by water was significantly different from that extracted by 70% methanol. EtAc showed the worst ability to extract polyphenols and flavonoids, which is consistent with the finding of Wang et al. [25], who also reported that EtAc had the worst extraction efficiency for antioxidants from partridge leaf-tea [25]. The antioxidant property of KDC extracts was clearly influenced by the type of DES extracted (Figure 1D). Pro-EthG (502.31 mmol TE/g DW), Pro-Gly (416.44 mmol TE/g DW) extracts and Bet-Gly (356.10 mmol TE/g DW) exhibited stronger ABTS^•+^ radical scavenging activities than others. The lowest ABTS^•+^ was observed in ChCl-LA extracts (107.58 mmol TE/g DW). Pro-Gly extract (185.19 mM Fe(II)E/g DW) showed the highest FRAP value, followed by Pro-EthG extract (163.48 μM Fe(II)E/g DW) and ChCl-LA extract (58.15 μM Fe(II)E/g DW) ranking the lowest. The ABTS^•+^ and FRAP values of Pro-Gly and Pro-EthG extracts were relatively higher. Moreover, the antioxidant activity of the KDC extracts using most of the DESs was significantly higher than activity when using conventional solvents. As we know, the physical properties of DESs, including polarity, pH value and viscosity, play an important role in the extraction process [15]. Figure 2 shows the correlation coefficient among the physical-chemical properties of DESs and the corresponding TPC, TFC, ABTS^•+^ and FRAP in the KDC extracts. The viscosity of DES was significantly negatively correlated with TPC and FRAP. A negative correlation was found between the polarity and viscosity of the extraction solvents (*r* = −0.52, *p* < 0.05). Researchers have confirmed that the viscosity of DESs significantly affects the cavitation and mass transfer efficiency in the process of ultrasonic extraction, thus affecting the extraction of phenolic compounds [26]. Moreover, there is a positive correlation between the polarity of DESs and TPC, ABTS^•+^ and FRAP (0.47 < *r* < 0.97, *p* < 0.05). In this study, DESs with high polarity and low viscosity have good extraction efficiency for active compounds of plant materials, which is also consistent with our previous work [15,27]. It is concluded that the antioxidant activity of the KDC extracts is positively related to the TFC. Therefore, Pro-Gly was screened as the best extractants.

### 3.2. Model Fitting and Response Surface Analysis

The effects of extraction time (A), extraction temperature (B), ultrasonic power (C), L/S (D) and water content of Pro-Gly (E) on TPC, TFC and antioxidant capacity (ABTS^•+^ and FRAP) were investigated by full-factorial design (FFD) experiments (Table S1). The results showed that A, B, D and E were significantly associated with TPC and TFC (*p* < 0.05), while the variable C was insignificantly related with TPC and TFC (*p* > 0.05). In addition, it was found that extraction temperature and water content of Pro-Gly (BE) had significant interaction influence on TFC. The extraction time and water content of Pro-Gly (AE) exerted interaction effects on TPC, TFC and FRAP (*p* < 0.05) (Figure S1A–D). 

According to the results of FFD experiments, extraction temperature (X_1_), extraction time (X_2_), L/S (X_3_) and water content of Pro-Gly (X_4_) were selected to further optimize TPC, TFC, ABTS^•+^ and FRAP by RSM (Figure S1A–D). Table 2 shows RSM design and predicted results for the optimization extraction of TPC, TFC, ABTS^•+^ and FRAP of the KDC extracts. The measured TPC, TFC, ABTS^•+^ and FRAP varied within 17.21−69.22 mg CAE/g DW, 16.58−91.90 mg RE/g DW, 111.67−525.86 mmol TE/g DW and 76.60−459.83 µM Fe(II)E/g DW, respectively, showing little deviation from the predicted values.

The second-order polynomial equations of TPC, TFC, ABTS^•+^ and FRAP are shown by Equations (2)–(5), respectively:

(2) $$\begin{array}{l} Y_{TPC}=50.72+9.47X_{1}+4.46X_{2}+0.6410X_{3}+10.52X_{4}+1.57X_{1}X_{2}+0.8354X_{1}X_{3}\\ -7.00X_{1}X_{4}-0.7377X_{2}X_{3}-1.82X_{2}X_{4}+0.9829X_{3}X_{4}-4.10X_{1}^{2}-1.50X_{2}^{2}\\ +2.59X_{3}^{2}-2.17X_{4}^{2} \end{array}$$

(3) $$\begin{array}{l} Y_{TFC}=75.93+15.88X_{1}+4.74X_{2}-0.0174X_{3}+14.75X_{4}+0.3376X_{1}X_{2}+0.9418X_{1}X_{3}\\ -10.00X_{1}X_{4}+0.8467X_{2}X_{3}-2.78X_{2}X_{4}+1.12X_{3}X_{4}-3.91X_{1}^{2}-1.22X_{2}^{2}\\ +1.41X_{3}^{2}-5.71X_{4}^{2} \end{array}$$

(4) $$\begin{array}{l} Y_{ABTS+}=408.42+82.59X_{1}+14.66X_{2}+16.19X_{3}+61.45X_{4}+6.92X_{1}X_{2}+19.21X_{1}X_{3}\\ -33.43X_{1}X_{4}+8.50X_{2}X_{3}-6.82X_{2}X_{4}+16.32X_{3}X_{4}-14.95X_{1}^{2}-7.41X_{2}^{2}\\ +3.66X_{3}^{2}-30.10X_{4}^{2} \end{array}$$

(5) $$\begin{array}{l} Y_{FRAP}=339.34+67.26X_{1}+21.53X_{2}+10.80X_{3}+66.19X_{4}+0.6177X_{1}X_{2}+8.27X_{1}X_{3}\\ -44.47X_{1}X_{4}+9.49X_{2}X_{3}-7.10X_{2}X_{4}-3.05X_{3}X_{4}-22.30X_{1}^{2}+1.53X_{2}^{2}\\ +5.20X_{3}^{2}-21.35X_{4}^{2} \end{array}$$

Table 3 shows the results of the ANOVA and regression coefficients. The results of the ANOVA indicated that the four models were of high significance (*p* < 0.0001), while the lack of fit of each model was insignificant (*p* > 0.05), indicating that the models well predicted the actual results. The correlation coefficient (R^2^) of TPC, TFC, ABTS^•+^ and FRAP were relatively high (0.8893, 0.9510, 0.9375 and 0.9136, respectively), indicating the consistency between measured and predicted values. The R^2^ and adjusted coefficient of determination (Adj. R^2^) were around 0.9, indicating a significant correlation between the measured and predicted values. In addition, the lower the coefficient of variation, the smaller the variation of the mean value, and the higher the precision and reliability of the experimental values. The effects of independent variables on response variables can be characterized by F-values and *p*-values of the linear and quadratic coefficients [28]. It was found that TPC was mainly affected by X_1_ and X_4_, followed by X_2_, X_1_X_4_ and X_1_^2^. Linear X_1_ and X_4_ coefficients exhibited a significant effect (*p* < 0.01) on TPC, while X_1_^2^ exhibited a significant influence (*p* < 0.05) on TPC. The water content in Pro-Gly and extraction temperature (X_1_X_4_) exhibited an evident interaction effect on TPC. TFC was affected by X_1_, X_4_, followed by X_2_, X_1_X_4_ and X_1_^2^; X_4_^2^. X_1_, X_2_ and X_4_ coefficients had a significant effect (*p* < 0.01) on TFC; and X_1_^2^ and X_4_^2^ had a significant (*p* < 0.05) and extremely significant effect (*p* < 0.001) on TFC, respectively. However, X_1_X_4_ exhibited a significant interaction effect on TFC. In terms of antioxidant activity, the effect of independent variables on the response values in ABTS^•+^ was roughly the same as that in TFC. ABTS^•+^ value was greatly influenced by X_1_ and X_4_, followed by X_1_X_4_, X_1_^2^ and X_4_^2^. Linear X_1_ and X_4_ coefficients had an extremely significant effect (*p* < 0.001) on ABTS^•+^, while the X_1_^2^ and X_4_^2^ coefficients had a significant (*p* < 0.05) and extremely significant (*p* < 0.001) effect on ABTS^•+^, respectively. Ultrasound temperature and extraction time (X_1_X_4_) exhibited a significant interaction effect on ABTS^•+^. FRAP was greatly affected by X_1_, X_2_ and X_4_, followed by X_1_X_4_, X_1_^2^ and X_4_^2^. Linear X_1_, X_4_ coefficients had highly significant (*p* < 0.01) effects on FRAP, while the X_1_^2^ and X_1_^2^ had significant effects (*p* < 0.05) on FRAP. Extraction temperature and L/S (X_1_X_4_) exhibited an extremely significant interaction effect on FRAP (*p* < 0.001). 

**Figure 3:**
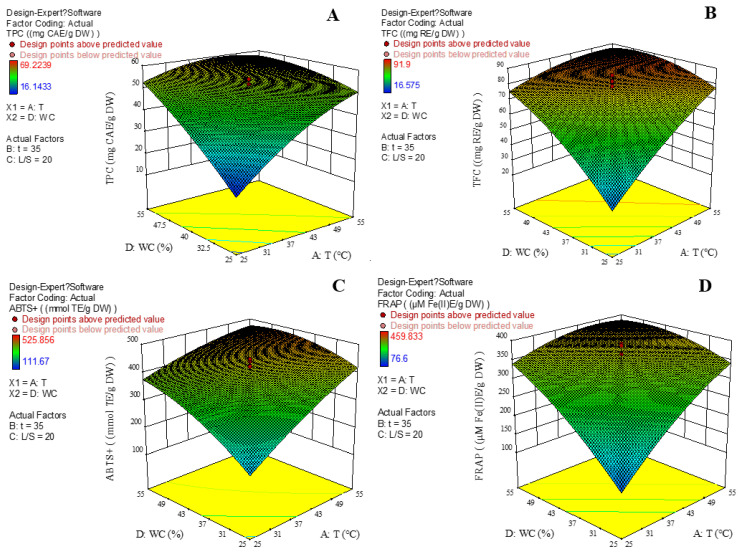
Interaction effects between the independent variables on TPC (**A**), TFC (**B**), ABTS^•+^ (**C**) and FRAP (**D**) of the KDC extracts. TPC, total phenolic content; TFC, total flavonoid content; WC, water content in Pro-Gly; L/S, liquid to solid ratio; T, extraction temperature; t, extraction time.

Figure 3 shows the 3D response surface plots for RSM. It is clear that water content in DES was a key variable affecting TPC, TFC and the antioxidant activities. It was revealed that TPC, TFC, ABTS^•+^ and FRAP of the KDC extracts increased with X_1_. Figure 3 shows that TPC, TFC, ABTS^•+^ and FRAP of the KDC extracts were increased when the water content in DES was increased up to 55%, which was consistent with Wu et al. [29]. Therefore, water content in DES is the dominant factor affecting the extraction of phenolics from KDC [29].

### 3.3. Validation of Optimization of Pro-Gly-Based UAE Process

To validate the integrity and feasibility of the RSM design model, the confirmation experiment was carried out under the optimal parameters of UAE obtained. Through 3D surface analysis, the optimal conditions for concurrently improving the TPC, TFC, as well as antioxidant activities of the KDC extracts are as follows: 46.4% of water content in Pro-Gly; extraction temperature of 55 °C; L/S of 25:1 (mL/g); and 50 min of ultrasonic time. Under optimal conditions, TPC, TFC, ABTS^•+^ and FRAP were measured to be 69.31 ± 1.58 mg CAE/g DW, 96.03 ± 1.13 mg RE/g DW, 546.30 ± 1.30 mmol TE/g DW and 451.22 ± 4.44 µmol Fe(II)/g DW, respectively. In contrast, the predicted values were 64.701 mg CAE/g DW of TPC; 95.219 mg RE/g DM for TFC; 548.320 mmol TE/g DW for ABTS^•+^; and 442.778 μmol Fe(II)SE/g DW for FRAP. The deviation between measured and predicted results was low, indicating the feasibility of the model.

### 3.4. Identification of Phenolic Composition

Table 4 shows the tentative identification results of chemical constituents in the KDC extract based on their mass fragmentation pattern. Peak 1 (RT 5.03 min) was easily identified as chlorogenic acid considering its parent ion at *m*/*z* 353.09 [C_16_H_18_O_9_-H]^−^ and the retention time in HPLC chromatograms. Peak 2 (RT 6.25 min), which showed a parent ion *m*/*z* of 305.06 [C_15_H_14_O_7_-H]^−^ and MS^2^ fragments ions at *m*/*z* of 219.10 and 177.01, was identified as epigallocatechin. Peak 3 (RT 6.62 min) with the parent ion at *m*/*z* of 447.38 [C_21_H_20_O_11_-H]^−^, MS^2^ fragments ions at *m*/*z* 286.15 [C_15_H_10_O_6_-H]^−^ and *m*/*z* 159.51 was temporarily identified as kaempferol 3-*O*-*β*-glucopyranoside [30]. Peak 4 was easily determined as kaempferol 3-rutinoside based on the parent ion at *m*/*z* 593.16 [C_27_H_30_O_15_-H]^−^ and the fragment ions at *m*/*z* 285.13 [C_15_H_10_O_6_-H]^−^ were indicative of the presence of kaempferol and *m*/*z* 159.51. Peaks 5 and 6 showed the same molecular ion at *m*/*z* 353.09 [C_16_H_18_O_9_−H]^−^ were determined as 5-caffeoylquinic acid and 3-caffeoylquinic acid, respectively. Peak 7 (RT 14.87 min) was determined as dihydroquercetin due to its parent ion at *m*/*z* 305.26 [C_15_H_12_O_7_+H]^+^ [31]. Peak 9 (RT 18.17 min), 11 (RT 21.03 min) and 12 (RT 22.87 min) had identical molecular ion at *m*/*z* 515.47 [C_25_H_24_O_12_-H]^−^ and fragment ions at *m*/*z* of 353.09, 191.06 and 185.02, which were identified as 3,5-dicaffeoylquinic acid, 3,5-dicaffeoylquinic acid and 4,5-dicaffeoylquinic acid, respectively. Peak 13 (RT 31.38 min), indicating the parent ion at *m*/*z* 320.60 [C_15_H_10_O_8_+H]^+^ and its fragment ions at *m*/*z* 273.10 and 179.21, was easily determined as myricetin [32]. Peak 15, with a molecular formula of C_16_H_12_O_7_, was easily identified as isorhamnetin. In addition, peaks 8 and 14 cannot be currently identified based on the available information. 

### 3.5. UAE–Pro-Gly Method and Other Extraction Methods

**Figure 4:**
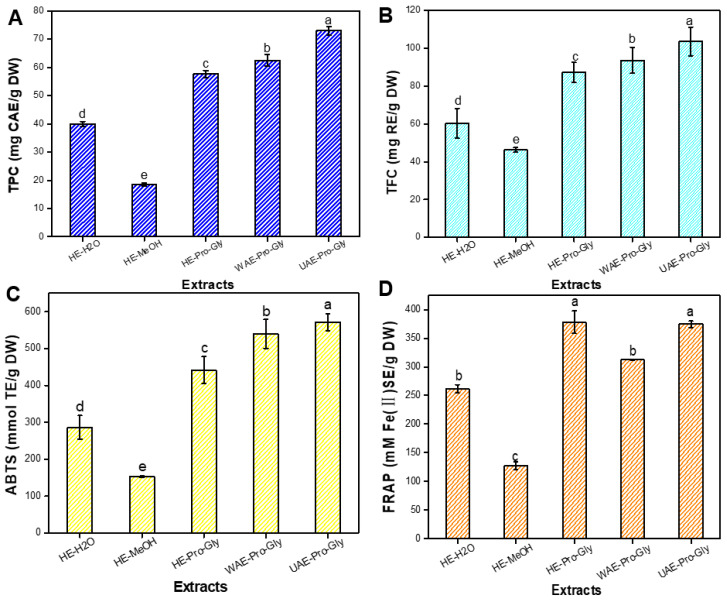
Comparative TPC (**A**), TFC (**B**), ABTS^•+^ (**C**) and FRAP (**D**) in the KDC extracts extracted by different methods. Different lowercase letters (a–e) in the figures mean significant difference (*p* < 0.05).

Figure 4A–D show that the UAE–Pro-Gly extract exhibited the highest TPC, TFC, ABTS^•+^ and FRAP. HE–Pro-Gly extract showed higher TPC, TFC, ABTS^•+^ and FRAP values than the HE–H_2_O extract and HE–MeOH extract. By contrast, the MWE–Pro-Gly extract showed slightly higher TPC, TFC and ABTS^•+^ than the HE–Pro-Gly extract, except for FRAP. The results implies that the Pro-Gly as extractants combined with UAE procedure is a green approach for extracting phenolic antioxidants from KDC.

**Figure 5:**
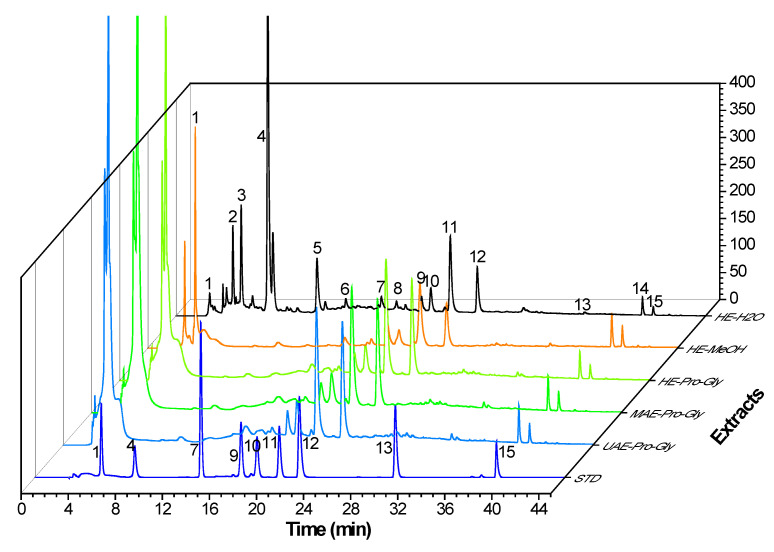
HPLC profiles of the KDC extracts extracted by different methods. STD, standards; 1, 5- Chlorogenic acid; 4, Kaempferol-3-rutinoside; 7, dihydroquercetin; 9, 3,4-Dicaffeoylquinic acid; 10, Quercetagetin; 11, 3,5-Dicaffeoylquinic acid; 12, 4,5-Dicaffeoylquinic acid; 13, Myricetin; 15, Isorhamnetin.

The HPLC profiles of the KDC extracts under various extraction methods are shown in Figure 5. Major extracted phenolics were quantified by HPLC-DAD. Table 5 shows the content of individual phenolics in the KDC extracts extracted by different methods. The results confirmed that the contents of individual phenolics were greatly affected by extraction solvents and methods. Significant differences can be observed in individual phenolic content under HE using water, MeOH and Pro-Gly as solvents. A high content of 3,4-dicaffeoylquinic acid was detected in HE–H_2_O extracts. 5-chlorogenic acid could hardly be detected in the HE–MeOH extract. Higher content of flavonoids and dicaffeoylquinic acid compounds were found in the HE–MeOH and HE–Pro-Gly extracts. Compared to extraction using water and MeOH, Pro-Gly had a wide polarity and excellent solubility for active molecules of KDC, thus effectively improving the extraction of active compounds [33,34]. It can be seen that the UAE–Pro-Gly extract contained high levels of phenolic compounds, including 5-chlorogenic acid (11,418.35 ± 66.38 μg/g DW), followed by 4,5-dicaffeoylquinic acid (5942.71 ± 369.04 μg/g DW), myricitrin (5509.43 ± 327.87 μg/g DW), 3,5-dicaffeoylquinic acid (2257.49 ± 179.42 μg/g DW), 3,4-dicaffeoylquinic acid (1356.78 ± 58.45 μg/g DW), isorhamnetin (984.63 ± 15.94 μg/g DW) and kaempferol 3-rutinoside (1241.87 ± 43.44 μg/g DW). MAE–Pro-Gly contained higher amounts of 5-chlorogenic acid as compared to UAE–Pro-Gly. However, 3,4-dicaffeoylquinic acid could not be detected in the MAE–Pro-Gly extract. Compared to HE, UAE/MAE greatly enhanced the extraction of phenolics from KDC. Researchers have verified that ultrasound can enhance mass and energy-transfer during extraction, thus improving the releasing and extraction of phytochemicals from natural products [35,36]. Currently, many innovative extraction methods combined with DESs have been developed and are widely applied [37,38]. Wang et al. [29] found that ultrasound combined with a novel DES synthesized by choline chloride and malic acid exhibited excellent performance in extracting antioxidants from partridge leaf-tea. Fu et al. [39] confirmed that sonication-synergistic ChCl-based DES facilitated the structure destruction of *Carya cathayensis* peel, leading to the release of more phytochemicals from plant materials. In this study, UAE–Pro-Gly indicated a high efficiency in extracting the phenolic compounds from KDC, which is in agreement with Fu et al. [39]. Therefore, Pro-Gly coupled with UAE is an ideal approach for extracting phenolics from KDC. 

### 3.6. SEM

**Figure 6:**
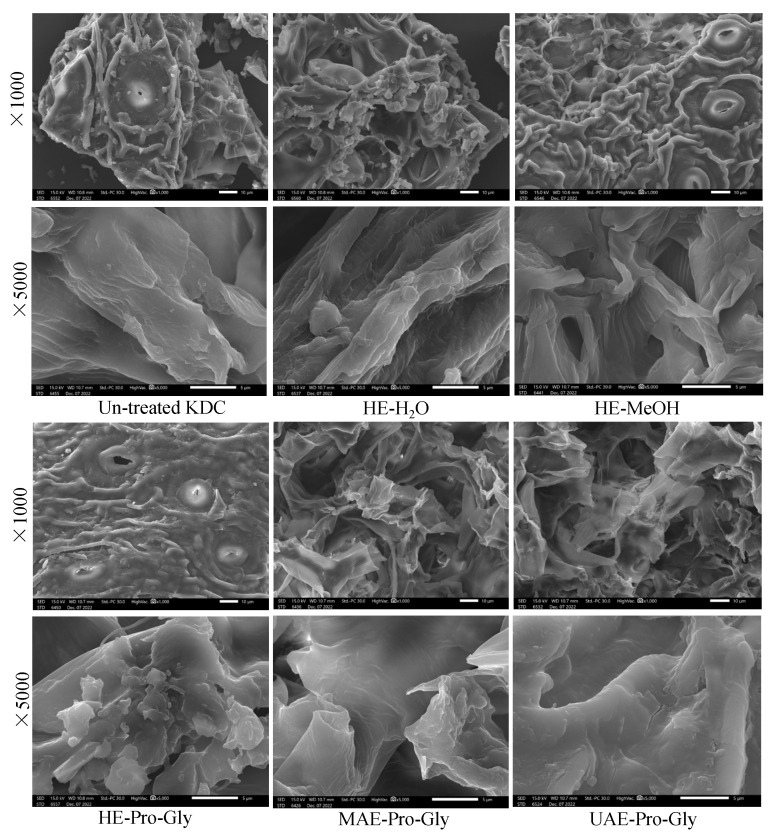
SEM analysis of the untreated KDC and the KDC remnants after extraction using different solvents.

Figure 6 shows the effect of different extraction methods on the surface structure morphology of the KDC residues after extraction. Compared to untreated KDC raw, different extraction solvents/methods resulted in significant changes in the microscopic morphology of plant cell wall structure. The morphology surface of sample prior to extraction is relatively lumpy and thick. After KDC sample treatment with water, methanol and Pro-Gly, the surface morphologies of the samples appeared to change to some extent. Among them, the morphology surface of the samples treated with water and MeOH became significantly loose, showing obvious pores and cracks. However, the KDC samples treated with Pro-Gly showed more visible pores, fissures and chasms, and the external surface was relatively thinner and transparent compared with the untreated KDC raw, which might be due to the partial erosion and penetration impacts of Pro-Gly on plant cell walls; thereby, more secondary metabolites from the plant matrix were released [39,40]. Thus, the extract obtained using Pro-Gly showed higher contents of active compounds than those obtained by using other solvents. Particularly, by comparison with other extraction methods, the surface morphologies of the KDC samples treated with MAE–Pro-Gly or UAE–Pro-Gly were significantly damaged, with more obvious pores and cracks. This may be due to the fact that ultrasound cavitation, erosion and penetration impacts of Pro-Gly have synergistic effects on the destruction of plant cell wall structures, which agrees well with the results of Huang et al. [16]. In addition, Wang et al. [25] also reported that DESs can improve the solubilization, transport and releasing of poorly water-soluble metabolites in plant cellular tissues, thereby obtaining more active compounds in the KDC extract.

## 4. Conclusions

In the present work, a novel, eco-friendly and highly efficient DES-based UAE method was developed to extract antioxidants from KDC. The best solvent consisting of L-proline and glycerol (Pro-Gly) was selected for extracting natural antioxidants from KDC. The optimal extraction parameters of UAE using Pro-Gly as the extraction solvent were obtained under an extraction temperature of 55 °C, extraction time of 50 min, L/S of 25:1 and water content in Pro-Gly of 46.4% using RSM. In addition, 5-chlorogenic acid, 4,5-dicaffeoylquinic acid, 3,5-dicaffeoylquinic acid, 3,4-dicaffeoylquinic acid, kaempferol 3-rutinoside, myricetin and isorhamnetin were found to be the major phytochemicals in the KDC extract. UAE–Pro-Gly yielded higher individual phenolics contents, TPC, TFC, ABTS^•+^ and FRAP than other extraction methods. In short, UAE–Pro-Gly can be considered as a promising method for extracting natural antioxidants from KDC, which can be used in food, cosmetic and pharmaceutical industries. 

## Figures and Tables

**Table 1:** Lists and physicochemical properties of deep eutectic solvents (DESs) prepared in this study.

No.	Component A	Component B	Abbreviations	Molar Ratio (mol/mol)	pH	Viscosity (mPa·s, 30% Water)	Polarity
1	Choline chloride	Levulinic acid	ChCl-LevA	1:2	1.35	571.47	49.29
2	Choline chloride	Fructose	ChCl-Fru	1:1	3.56	480.84	48.87
3	Choline chloride	Sucrose	ChCl-Suc	1:1	7.00	576.06	49.31
4	Choline chloride	Malic acid	ChCl-MA	1:1	5.86	522.21	49.74
5	Choline chloride	Citric acid	ChCl-CA	1:2	5.16	620.74	48.87
6	Choline chloride	Glucose	ChCl-Glu	1:1	5.24	305.74	48.86
7	Choline chloride	Xylitol	ChCl-Xyl	1:1	3.73	609.52	49.72
8	Choline chloride	Glycerol	ChCl-Gly	1:2	3.90	445.85	49.71
9	Choline chloride	Lactic acid	ChCl-LA	1:2	3.07	717.39	48.87
10	Choline chloride	Ethylene glycol	ChCl-EthG	1:2	3.98	266.99	49.73
11	Betaine	Glycerol	Bet-Gly	1:2	2.47	701.19	48.46
12	Betaine	Levulinic acid	Bet-LevA	1:2	6.62	673.39	49.29
13	Betaine	Malic acid	Bet-MA	1:1	6.99	541.12	49.29
14	L-proline	Glycerol	Pro-Gly	1:2	5.32	265.28	62.15
15	L-proline	Levulinic acid	Pro-LevA	1:2	3.76	582.56	49.76
16	L-proline	Ethylene glycol	Pro-EthG	1:2	7.18	533.73	49.70

**Table 2:** RSM design and predicted results for the optimization of TPC, TFC, ABTS^•+^ and FRAP.

Factors			Areb.	Unit	Actual Levels							
					−2	−1	0		1		2	
Extraction temperature			X_1_	℃	10	25	40		55		70	
Extraction time			X_2_	min	5	20	35		50		65	
Liquid to solid ratio (L/S)			X_3_	mL/g	10	15	20		25		30	
Water content in Pro-Gly			X_4_	*v*/*w*	10	25	40		55		70	
Run.	X_1_	X_2_	X_3_	X_4_	TPC		TFC		ABTS^•+^		FRAP	
					Exp.	Pred.	Exp.	Pred.	Exp.	Pred.	Exp.	Pred.
1	25	50	15	25	22.45	25.16	33.24	34.29	196.48	207.57	150.18	137.45
2	40	35	20	40	54.84	50.72	64.15	75.93	365.58	408.42	269.64	339.34
3	40	35	20	40	44.19	50.72	64.15	75.93	352.62	408.42	283.94	339.34
4	70	35	20	40	39.93	53.25	91.90	92.03	525.86	513.79	373.27	384.64
5	40	35	20	40	49.56	50.72	78.48	75.93	422.08	408.42	339.20	339.34
6	40	35	20	70	57.08	63.09	86.98	82.57	453.13	410.92	420.09	386.30
7	25	20	15	25	17.21	14.28	28.84	21.61	256.50	195.44	142.31	100.39
8	40	35	10	40	55.10	59.81	82.08	81.60	384.81	390.71	347.90	338.55
9	40	5	20	40	23.68	35.78	59.15	61.58	327.91	349.46	294.31	302.38
10	25	20	25	25	17.39	13.40	19.31	15.77	143.52	139.74	92.00	92.57
11	40	65	20	40	54.85	53.62	80.40	80.55	418.40	408.11	385.42	388.51
12	55	50	15	55	66.04	61.02	81.74	86.54	406.40	424.76	389.23	380.92
13	55	50	25	55	63.40	64.47	88.94	92.32	510.00	545.21	393.44	431.94
14	55	20	15	55	54.78	51.11	87.74	83.63	415.04	412.25	382.57	369.81
15	55	20	15	25	50.71	42.41	65.49	70.80	360.62	375.23	293.40	306.07
16	55	50	15	25	60.63	59.59	90.49	84.82	439.05	415.03	339.23	345.60
17	55	20	25	55	69.22	57.51	85.81	86.02	495.19	498.68	377.89	382.87
18	10	35	20	40	17.81	15.37	26.08	28.53	160.12	183.44	115.82	115.61
19	40	35	20	40	52.81	50.72	85.65	75.93	439.37	408.42	391.79	339.34
20	25	50	15	55	54.74	54.61	75.86	75.99	352.10	351.04	329.73	350.65
21	25	20	25	55	54.88	54.05	71.23	73.05	377.61	375.79	331.78	321.99
22	40	35	20	10	16.14	21.01	16.58	23.57	111.67	165.14	76.60	121.55
23	25	20	15	55	52.91	51.00	70.36	74.43	344.41	366.20	298.73	342.01
24	55	50	25	25	66.20	59.10	88.94	86.13	477.39	470.18	459.83	408.82
25	55	20	25	25	46.61	44.87	72.69	68.72	421.16	396.37	355.67	331.33
26	25	50	25	25	19.53	21.33	31.55	31.82	208.95	185.89	158.25	167.59
27	25	50	25	55	55.42	54.71	82.06	78.00	394.68	394.65	389.00	368.59
28	40	35	30	40	56.20	62.37	78.48	81.53	450.09	455.46	361.24	381.75
29	40	35	20	40	55.02	50.72	81.48	75.93	422.24	408.42	364.68	339.34
30	40	35	20	40	47.92	50.72	81.65	75.93	448.65	408.42	386.76	339.34

**Table 3:** ANOVA for response surface quadratic model.

Term	Df	TPC		TFC		ABTS^•+^		FRAP	
		*F* Value	*p* Value	*F* Value	*p* Value	*F* Value	*p* Value	*F* Value	*p* Value
Mode	14	8.61 ***	<0.0001	20.80 ***	<0.0001	16.08 ***	<0.0001	11.33 ***	<0.0001
X_1_	1	36.53 ***	<0.0001	117.89 ***	<0.0001	112.65 ***	<0.0001	59.42 ***	<0.0001
X_2_	1	8.10 *	0.0123	10.53 **	0.0054	3.55	0.0790	6.09	0.0261
X_3_	1	0.17	0.6883	1.424 × 10^−4^	0.9906	4.33	0.0551	1.53	0.2349
X_4_	1	45.09	<0.0001	101.77 ***	<0.0001	62.36 ***	<0.0001	57.54 ***	<0.0001
X_1_X_2_	1	0.67	0.4248	0.036	0.8530	0.53	0.4792	3.341 × 10^−4^	0.9547
X_1_X_3_	1	0.19	0.6695	0.28	0.6066	4.06	0.0621	0.60	0.4510
X_1_X_4_	1	13.32 **	0.0024	31.16 ***	<0.0001	12.31 **	0.0032	17.32 ***	0.0008
X_2_X_3_	1	0.15	0.7061	0.22	0.6431	0.80	0.3863	0.79	0.3886
X_2_X_4_	1	0.90	0.3586	2.40	0.1418	0.51	0.4852	0.44	0.5163
X_3_X_4_	1	0.26	0.6160	0.39	0.5415	2.93	0.1073	0.081	0.7793
X_1_^2^	1	7.84 **	0.0135	8.18 *	0.0119	4.22 *	0.0478	7.47 *	0.0154
X_2_^2^	1	1.05	0.3209	0.79	0.3883	1.04	0.3248	0.035	0.8540
X_3_^2^	1	3.13	0.0973	1.06	0.3190	0.25	0.6219	0.41	0.5334
X_4_^2^	1	2.19	0.1598	17.45 ***	0.0008	17.10 ***	0.0009	6.84 *	0.0195
R^2^		0.8893		0.9510		0.9375		0.9136	
Adj R^2^		0.8860		0.9053		0.8792		0.8930	
Pre R^2^		0.7893		0.8394		0.8381		0.8872	
Adeq precision		9.408		15.114		15.042		11.228	
Lack of fit (*F*-value)	10	4.32		0.37		0.88		0.51	
Lack of fit (*p*-value)		0.6100 ^ns^		0.9148 ^ns^		0.5985 ^ns^		0.8293 ^ns^	

ns. not significant (*p* > 0.05). * Significant at (*p* < 0.05). ** Highly significant at (*p* < 0.01). *** Extremely significant at (*p* < 0.001).

**Table 4:** Identification of bio-active compositions in the KDC extract.

No.	Retention Time (min)	λ_max_ (nm)	Molecular Ion (*m*/*z*)	MS Ion Fraction (*m*/*z*)	Mw	Formula	Compounds	Error	References
1	5.03	254, 280	353.09 [M-H]^−^	353.09, 191.06, 185.05	354	C_16_H_18_O_9_	Chlorogenic acid	0.6	Standard, MS/MS
2	6.25	256, 350	305.02 [M-H]^−^	305.02, 219.10, 177.01	306	C_15_H_14_O_7_	Epigallocatechin	0.2	MS/MS
3	6.62	256, 350	447.38 [M-H]^−^	447.38, 285.08, 159.51	448	C_21_H_20_O_11_	Kaempferol 3-O-β-glucopyranoside	1.3	MS/MS
4	8.78	254, 353	593.16 [M-H]^−^	593.16, 285.13, 159.52	594	C_27_H_30_O_15_	Kaempferol 3-rutinoside	−0.4	Standard, MS/MS
5	12.05	255, 280	354.09 [M-H]^−^	353.09, 191.06, 185.02	354	C_16_H_18_O_9_	5-Caffeoylquinic acid	−0.4	MS/MS
6	12.49	255, 280	354.09 [M-H]^−^	353.09, 191.06, 185.05	354	C_16_H_18_O_9_	3-Caffeoylquinic acid	−0.5	MS/MS
7	14.87	254, 354	305.26 [M+H]^+^	304.26, 303.05	304	C_15_H_12_O_7_	Dihydroquercetin	−2.3	Standard, MS/MS
8	15.36	254, 350	433.15 [M+H]^+^	433.15, 270.24, 161.09	432	C_21_H_20_O_10_	Unidentifed	3.7	-
9	18.17	254, 280	515.47 [M-H]^−^	515.47, 353.09, 191.06, 185.02	516	C_25_H_24_O_12_	3,4-Dicaffeoylquinic acid	0.9	Standard, MS/MS
10	19.09	254, 350	317.24 [M-H]^−^	318.24, 302.06	318	C_15_H_10_O_8_	Quercetagetin	−0.3	Standard, MS/MS
11	21.03	254, 280	515.47 [M-H]^−^	515.47, 353.09, 191.06, 185.02	516	C_25_H_24_O_12_	3,5-Dicaffeoylquinic acid	0.6	Standard, MS/MS
12	22.87	255, 280	515.47 [M-H]^−^	515.47, 353.09, 191.06, 185.02	516	C_25_H_24_O_12_	4,5-Dicaffeoylquinic acid	1.8	Standard, MS/MS
13	31.38	254, 350	319.60 [M+H]^+^	320.60, 319.60, 273.10, 179.21	318	C_15_H_10_O_8_	Myricetin	0.4	Standard, MS/MS
14	37.65	254, 280	367.10 [M-H]^−^	367.10, 161.18, 135.12, 133.06	368	-	Unidentifed	3.2	-
15	39.02	254, 352	317.17 [M+H]^+^	317.17, 230.13, 154.21	316	C_16_H_12_O_7_	Isorhamnetin	−1.8	Standard, MS/MS

**Table 5:** The content of the main phenolic components in the KDC extracts obtained by different methods.

Compounds	Contents (μg/g DW)				
	HE–H_2_O	HE–MeOH	HE–Pro-Gly	MAE –Pro-Gly	UAE–Pro-Gly
5-Chlorogenic acid	10,330.98 ± 63.64 b	960.73 ± 13.59 a	10,191.55 ± 140.17 b	12,319.03 ± 116.56 d	11,418.35 ± 66.38 c
Kaempferol-3-rutinoside	400.47 ± 36.47 a	816.36 ± 58.70 b	1103.27 ± 28.63 c	1081.58 ± 39.24 c	1241.87 ± 43.44 d
dihydroquercetin	179.91 ± 20.08 a	180.94 ± 30.96 a	182.82 ± 10.44 a	191.49 ± 20.63 b	190.79 ± 13.97 b
3,4-Dicaffeoylquinic acid	1326.78 ± 290.99 c	320.48 ± 15.31 b	293.68 ± 48.91 a	ND.	272.32 ± 5.89 a
Quercetagetin	771.04 ± 29.73 a	1196.40 ± 23.35 b	1176.76 ± 32.74 b	1305.72 ± 34.95 c	1356.78 ± 58.45 c
3,5-Dicaffeoylquinic acid	1022.13 ± 164.95 a	1455.26 ± 24.40 b	1947.51 ± 66.44 c	2228.11 ± 157.92 d	2257.49 ± 79.42 d
4,5-Dicaffeoylquinic acid	2575.13 ± 93.77 a	3699.77 ± 46.43 b	5344.94 ± 65.02 c	5749.81 ± 84.00 d	5942.71 ± 69.04 e
Myricetin	1752.04 ± 109.19 a	2373.87 ± 37.96 b	4622.16 ± 449.53 c	5361.44 ± 82.56 d	5509.43 ± 37.87 e
Isorhamnetin	481.04 ± 109.54 a	928.44 ± 24.43 c	758.55 ± 41.42 b	927.43 ± 66.38 c	984.63 ± 15.94 d

ND.; not detected; MeOH, methanol; HE, heat extraction; MAE, microwave-assisted extraction; UAE, ultrasonic-assisted extraction. Different lowercase letters (a–e) in same row mean significant difference (*p* < 0.05).

## Data Availability

Data are contained within the article.

## Supplementary Materials

The following are available online at https://www.mdpi.com/article/10.3390/foods12091872/s1, Figure S1: The normal plot (A–D) of obtained from two-level factorial experiment showing the significance of the primary and interaction effects, Factor A, extraction time; Factor B, ultrasonic temperature; Factor C, ultrasonic power (P); Factor D, liquid-solid ratio (L/S); Factor E, water content in DES (WC); Table S1: FFD design and predicted results for the optimization of TPC, TFC, ABTS^•+^ and FRAP.
