# Supplementary material for: Deep Eutectic Solvents-Based Ultrasound-Assisted Extraction of Antioxidants from Kudingcha (llex kudingcha C.J. Tseng): Process Optimization and Comparison with Other Methods
Source: Foods. 2023 Apr 30;12(9):1872. doi: 10.3390/foods12091872 (PMC10178550; doi:10.3390/foods12091872)
Supplement: Supplementary file 1 [file foods-12-01872-s001.zip › foods-2350701-supplementary.pdf]

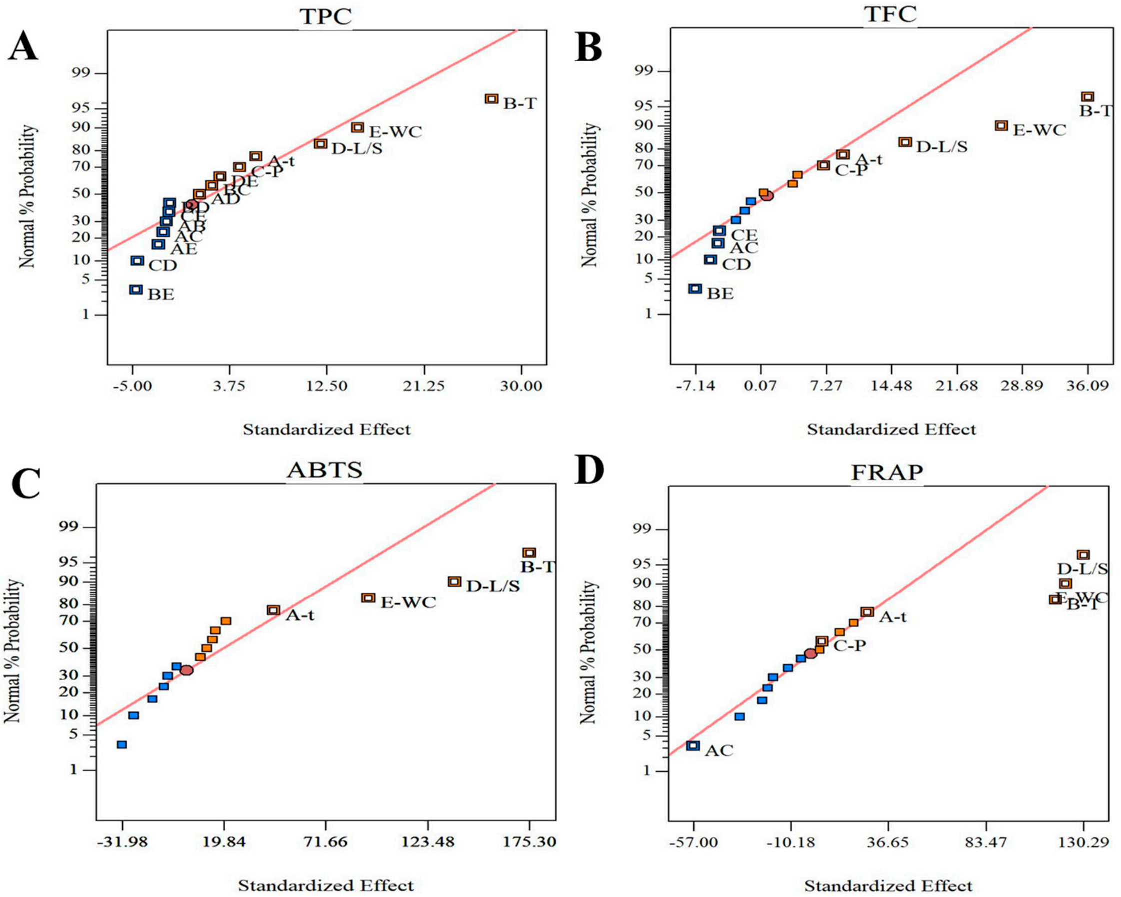

Figure S1: The normal plot (A-D) of obtained from two-level factorial experiment showing the significance of the primary and interaction effects, Factor A, extraction time; Factor B, ultrasonic temperature; Factor C, ultrasonic power (P); Factor D, liquid-solid ratio (L/S); Factor E, water content in DES (WC).

Table S1. FFD design and predicted results for the optimization of TPC, TFC, $ABTS^{\bullet +}$ and FRAP.

Factors				Areb.	Unit	Experimental value			
						-1		+1	
Extraction time				X_1	min	20		60	
Extraction temperature				X_2	°C	20		50	
Ultrasonic power				X_3	W	200		400	
Liquid to solid ratio (L/S)				X_4	mL/g	10		30	
Water content in Pro-Gly				X_5	*v*/*w*	20		40	
Run.	X_1	X_2	X_3	X_4	X_5	TPC	TFC	ABTS	FRAP
1	20	20	200	10	40	20.32	28.50	166.29	131.01
2	60	50	200	10	40	47.09	68.33	329.63	270.64
3	60	50	200	30	20	53.34	64.61	448.86	338.80
4	60	20	400	30	20	24.48	27.11	218.96	163.13
5	20	50	400	30	20	49.40	65.86	414.73	334.47
6	60	20	200	10	20	10.80	8.91	104.28	62.71
7	60	50	400	10	20	51.12	65.45	313.41	136.58
8	20	20	400	10	20	9.90	10.29	86.15	55.90
9	20	20	400	30	40	38.74	53.36	309.12	253.13
10	20	50	200	10	20	32.34	36.29	211.52	98.99
11	20	50	400	10	40	56.30	74.41	354.43	308.12
12	60	50	400	30	40	64.43	87.70	501.46	353.13
13	20	50	200	30	40	61.09	79.61	448.80	389.467
14	60	20	400	10	40	29.99	41.74	206.94	177.27
15	20	20	200	30	20	13.11	12.99	149.98	84.80
16	60	20	200	30	40	48.95	70.61	378.67	366.58
